# Supplementary material for: Spatial sampling in human visual cortex is modulated by both spatial and feature-based attention
Source: eLife. 2018 Dec 7;7:e36928. doi: 10.7554/eLife.36928 (PMC6286128; doi:10.7554/eLife.36928)
Supplement: Supplementary file 1. [file elife-36928-supp1.pdf]

# Supplementary tables

## Tables supporting main text and figures

**Table 1.** Statistics corresponding to Figure 2B, detailing the slope between pRF size and eccentricity across the different ROIs. P-values reflect whether the bootstrapped distribution is different from 0. Triple asterisks indicate FDR corrected significance of <.001. FDR test performed over all p-values in this table simultaneously.

| ROI | mean slope | p-value | N |
|---|---|---|---|
| V1 | 0.075 | <.001*** | 2176 |
| V2 | 0.122 | <.001*** | 2754 |
| V3 | 0.197 | <.001*** | 2322 |
| hV4 | 0.328 | <.001*** | 1201 |
| VO | 0.271 | <.001*** | 582 |
| LO | 0.290 | <.001*** | 1405 |
| V3AB | 0.202 | <.001*** | 883 |
| IPS0 | 0.299 | <.001*** | 316 |
| MT+ | 0.606 | <.001*** | 328 |

**Table 2.** Statistics corresponding to Figure 3C on pRF shift direction ratios. P-values reflect whether bootstrapped distribution is different from 0. Single, double and triple asterisks indicate FDR corrected significance of <.05, <.01 and <.001 respectively. FDR test performed over all p-values in this table simultaneously.

| | x>y | | | | ecc>x | | | |
|---|---|---|---|---|---|---|---|---|
| ROI | N | p | Δ ratio | Cohen's d | N | p | Δ ratio | Cohen's d |
| V1 | 2176 | .098 | .02 | .04 | 2176 | <.001*** | .03 | .09 |
| V2 | 2752 | .102 | .02 | .03 | 2752 | .002** | .02 | .06 |
| V3 | 2320 | .773 | .00 | .01 | 2318 | <.001*** | .07 | .18 |
| hV4 | 1201 | <.001*** | .10 | .18 | 1201 | <.001*** | .07 | .20 |
| VO | 582 | <.001*** | .34 | .67 | 573 | <.001*** | .08 | .37 |
| LO | 1397 | <.001*** | .12 | .20 | 1394 | <.001*** | .06 | .16 |
| V3AB | 880 | <.001*** | .16 | .27 | 873 | <.001*** | .15 | .46 |
| IPS0 | 313 | .023* | .08 | .14 | 313 | <.001*** | .16 | .61 |
| MT+ | 325 | <.001*** | .36 | .68 | 306 | <.001*** | .04 | .28 |
| combined | 11946 | <.001*** | .06 | .10 | 11940 | <.001*** | .05 | .15 |

**Table 3.** Statistics corresponding to Figure 3 on uniformity of polar angle distributions. P-values test whether pRFs are distributed non-uniformly over polar angle (Rayleigh test). Triple asterisks indicate FDR corrected significance of <.001. FDR test performed over all p-values in this table simultaneously.

| ROI | N | z | p |
|---|---|---|---|
| V1 | 2176 | 66.848 | <.001*** |
| V2 | 2754 | 51.904 | <.001*** |
| V3 | 2322 | 63.704 | <.001*** |
| hV4 | 1201 | 50.418 | <.001*** |
| VO | 582 | 116.563 | <.001*** |
| LO | 1405 | 40.995 | <.001*** |
| V3AB | 883 | 32.554 | <.001*** |
| IPS0 | 316 | 56.946 | <.001*** |
| MT+ | 328 | 119.052 | <.001*** |
| combined | 11967 | 463.382 | <.001*** |

**Table 4.** Statistics corresponding to Figure 4A on pRF eccentricity changes. P-values reflect whether bootstrapped distribution is different from 0, for each ROI and each eccentricity bin (bin 3 and 4 in table below). Single, double and triple asterisks indicate FDR corrected significance of <.05, <.01 and <.001 respectively. FDR test performed over all p-values in this table simultaneously.

| ecc bin | 1 | | | | 2 | | | |
|---|---|---|---|---|---|---|---|---|
| ROI | N | p | Δ ecc | Cohen's d | N | p | Δ ecc | Cohen's d |
| V1 | 509 | <.001*** | -.044 | -.44 | 749 | <.001*** | -.016 | -.20 |
| V2 | 862 | 0.504 | -.002 | -.02 | 931 | .204 | .004 | .04 |
| V3 | 920 | <.001*** | .056 | .49 | 747 | <.001*** | .059 | .58 |
| hV4 | 693 | <.001*** | .109 | .67 | 359 | <.001*** | .194 | .64 |
| VO | 313 | <.001*** | .389 | 1.04 | 162 | <.001*** | .352 | .85 |
| LO | 1047 | <.001*** | .121 | .62 | 263 | <.001*** | .295 | .60 |
| V3AB | 220 | <.001*** | .600 | .86 | 348 | <.001*** | .623 | .87 |
| IPS0 | 170 | <.001*** | .473 | .91 | 86 | <.001*** | .876 | 1.05 |
| MT+ | 186 | <.001*** | 1.015 | .68 | 103 | <.001*** | .759 | .66 |
| combined | 4920 | <.001*** | .091 | .45 | 3748 | <.001*** | .065 | .37 |

**Table 5.** Statistics corresponding to Figure 4A on pRF eccentricity changes. P-values reflect whether bootstrapped distribution is different from 0, for each ROI and each eccentricity bin (bin 1 and 2 in table

above). Single, double and triple asterisks indicate FDR corrected significance of <.05, <.01 and <.001 respectively. FDR test performed over all p-values in this table simultaneously.

| ecc bin | 3 | | | | 4 | | | |
|---|---|---|---|---|---|---|---|---|
| ROI | N | p | Δ ecc | Cohen's d | N | p | Δ ecc | Cohen's d |
| V1 | 595 | <.001*** | -.028 | -.30 | 323 | .010* | -.019 | -.15 |
| V2 | 616 | <.001*** | -.017 | -.16 | 343 | <.001*** | -.057 | -.36 |
| V3 | 427 | <.001*** | .069 | .43 | 226 | <.001*** | -.050 | -.24 |
| hV4 | 110 | <.001*** | .313 | .91 | 39 | 0.046 | .115 | .27 |
| VO | 71 | <.001*** | .455 | 1.05 | 36 | .325 | .081 | .14 |
| LO | 56 | <.001*** | .434 | .64 | 31 | .260 | .108 | .17 |
| V3AB | 199 | <.001*** | .657 | .93 | 113 | .017* | .102 | .21 |
| IPS0 | 36 | <.001*** | .896 | 1.01 | 21 | .470 | .135 | .17 |
| MT+ | 18 | <.001*** | .818 | 1.04 | 18 | .249 | .240 | .27 |
| combined | 2128 | <.001*** | .043 | .22 | 1150 | <.001*** | -.027 | -.14 |

**Table 6.** Statistics corresponding to Figure 4B on pRF size changes. P-values reflect whether bootstrapped pRF size difference distribution is different from 0, for each ROI and eccentricity bins 1 and 2 (bin 3 and 4 in table below). Single, double and triple asterisks indicate FDR corrected significance of <.05, <.01 and <.001 respectively. FDR test performed over all p-values in this table simultaneously.

| ecc bin | 1 | | | | 2 | | | |
|---|---|---|---|---|---|---|---|---|
| ROI | N | p | Δ size | Cohen's d | N | p | Δ size | Cohen's d |
| V1 | 509 | .624 | .001 | .02 | 749 | <.001*** | .004 | .15 |
| V2 | 862 | .007* | -.006 | -.09 | 931 | .870 | .000 | .01 |
| V3 | 920 | .237 | .003 | .04 | 747 | .613 | -.001 | -.02 |
| hV4 | 693 | .040 | -.007 | -.08 | 359 | <.001*** | .021 | .24 |
| VO | 313 | <.001*** | .128 | .51 | 162 | <.001*** | .063 | .34 |
| LO | 1047 | .060 | .008 | .06 | 263 | <.001*** | .084 | .36 |
| V3AB | 220 | <.001*** | .268 | .76 | 348 | <.001*** | .189 | .65 |
| IPS0 | 170 | <.001*** | .501 | .99 | 86 | <.001*** | .369 | .79 |
| MT+ | 186 | <.001*** | .711 | .68 | 103 | <.001*** | .332 | .77 |
| combined | 4920 | <.001*** | .009 | .10 | 3748 | <.001*** | .010 | .16 |

**Table 7.** Statistics corresponding to Figure 4B on pRF size changes. P-values reflect whether bootstrapped pRF size difference distribution is different from 0, for each ROI and eccentricity bins 3 and 4 (bin 1 and 2 in table above). Single, double and triple asterisks indicate FDR corrected significance of <.05, <.01 and <.001 respectively. FDR test performed over all p-values in this table simultaneously.

| ecc bin | 3 | 4 |
|---|---|---|

| ROI | N | p | Δ size | Cohen's d | N | p | Δ size | Cohen's d |
|---|---|---|---|---|---|---|---|---|
| V1 | 595 | <.001*** | .010 | .33 | 323 | .663 | .001 | .03 |
| V2 | 616 | .012* | -.005 | -.10 | 343 | <.001*** | -.021 | -.31 |
| V3 | 427 | <.001*** | -.015 | -.19 | 226 | <.001*** | -.052 | -.54 |
| hV4 | 110 | .012* | .029 | .24 | 39 | <.001*** | -.114 | -.72 |
| VO | 71 | <.001*** | .066 | .41 | 36 | <.001*** | -.085 | -.98 |
| LO | 56 | .108 | -.026 | -.21 | 31 | .006** | -.119 | -.45 |
| V3AB | 199 | <.001*** | .075 | .37 | 113 | <.001*** | -.111 | -.62 |
| IPS0 | 36 | <.001*** | .260 | .78 | 21 | .988 | .003 | .01 |
| MT+ | 18 | .541 | .056 | .14 | 18 | .128 | .227 | .31 |
| combined | 2128 | .252 | -.002 | -.03 | 1150 | <.001*** | -.035 | -.38 |

**Table 8.** Statistics corresponding to Figure 4C on correlations between eccentricity and size changes. P-values are two-tailed tests whether bootstrapped distribution of pRF eccentricity and size change correlations across bins is different from 0. Triple asterisks indicate FDR corrected significance of <.001. FDR test performed over all p-values in this table simultaneously.

| ROI | R | N | p |
|---|---|---|---|
| V1 | 0.74 | 20 | <.001*** |
| V2 | 0.86 | 20 | <.001*** |
| V3 | 0.87 | 20 | <.001*** |
| hV4 | 0.87 | 20 | <.001*** |
| VO | 0.97 | 20 | <.001*** |
| LO | 0.95 | 20 | <.001*** |
| V3AB | 0.99 | 20 | <.001*** |
| IPS0 | 0.93 | 20 | <.001*** |
| MT+ | 0.98 | 20 | <.001*** |
| combined | 0.94 | 20 | <.001*** |

**Table 9.** Statistics corresponding to Figure 6D on feature-based attentional modulation. P-values are uncorrected two-tailed tests whether bootstrapped feature attentional modulation index distribution is different from 0. Double and triple asterisks indicate FDR corrected significance of <.01 and <.001 respectively. FDR test performed over all p-values in this table simultaneously.

| ROI | N | mean diff | p | Cohen's d |
|---|---|---|---|---|
| V1 | 2176 | 0.055 | <.001*** | 0.175 |
| V2 | 2752 | 0.045 | <.001*** | 0.149 |
| V3 | 2320 | 0.026 | <.001*** | 0.090 |
| hV4 | 1201 | 0.065 | <.001*** | 0.235 |
| VO | 582 | 0.068 | <.001*** | 0.235 |

| | | | | |
|---|---|---|---|---|
| LO | 1397 | 0.022 | 0.006** | 0.079 |
| V3AB | 880 | 0.098 | <.001*** | 0.334 |
| IPS0 | 313 | 0.129 | <.001*** | 0.396 |
| MT+ | 325 | 0.064 | <.001*** | 0.201 |
| comb | 11946 | 0.048 | <.001*** | 0.161 |

**Table 10.** Statistics corresponding to Figure 7 on correlation between feature preference and eccentricity. P-values reflect whether bootstrapped distribution of correlation value of feature preference and pRF eccentricity differs from 0. Single and triple asterisks indicate FDR corrected significance of <.05 and <.001 respectively. FDR test performed over all p-values in this table simultaneously.

| ROI | N | corr | p |
|---|---|---|---|
| V1 | 2154 | -0.191 | <.001*** |
| V2 | 2718 | -0.205 | <.001*** |
| V3 | 2274 | -0.189 | <.001*** |
| hV4 | 1169 | -0.199 | <.001*** |
| VO | 567 | -0.033 | 0.475 |
| LO | 1365 | -0.054 | 0.052 |
| V3AB | 877 | -0.315 | <.001*** |
| IPS0 | 313 | -0.139 | 0.012* |
| MT+ | 325 | -0.146 | 0.007* |

## Tables supporting supplementary figures

**Table 11.** Statistics corresponding to Figure 2 - figure supplement 1, detailing the slope between pRF size and eccentricity across the different ROIs. P-values result from t-tests over the 5 subject values. Single, double and triple asterisks indicate uncorrected p-value < .05, .01 and .001 respectively. The last column reflects in how many out of 5 subjects these differences were different from 0 with uncorrected bootstrapped p-values of <.05 over voxels.

| ROI | mean slope | p-value | N | sig in |
|---|---|---|---|---|
| V1 | 0.076 | 0.028* | 5 | 5 |
| V2 | 0.127 | 0.002** | 5 | 5 |
| V3 | 0.199 | 0.002** | 5 | 5 |
| hV4 | 0.336 | <.001*** | 5 | 5 |
| VO | 0.183 | 0.018** | 5 | 4 |
| LO | 0.298 | 0.001** | 5 | 5 |
| V3AB | 0.185 | 0.008** | 5 | 4 |
| IPS0 | 0.289 | 0.005** | 5 | 4 |
| MT+ | 0.572 | 0.015* | 5 | 4 |

**Table 12.** Statistics corresponding to Figure 3 - figure supplement 2, on pRF x versus y shift ratios. P-values result from t-tests over the 5 subject values. Single, double and triple asterisks indicate uncorrected p-value < .05, .01 and .001 respectively. The last two columns reflect in how many out of 5 subjects these differences were different from 0 with uncorrected bootstrapped p-values of <.05 over voxels.

| ROI | N | p | t | Δ ratio | Cohen's d | x>y | y<x |
|---|---|---|---|---|---|---|---|
| V1 | 5 | .614 | 0.546 | .02 | .27 | 2 | 1 |
| V2 | 5 | .638 | 0.508 | .02 | .25 | 2 | 2 |
| V3 | 5 | .882 | 0.158 | .01 | .08 | 1 | 2 |
| hV4 | 5 | .100 | 2.128 | .12 | 1.06 | 2 | 0 |
| VO | 5 | .004** | 5.858 | .35 | 2.93 | 5 | 0 |
| LO | 5 | .091 | 2.216 | .13 | 1.11 | 3 | 0 |
| V3AB | 5 | .165 | 1.699 | .14 | .085 | 3 | 0 |
| IPS0 | 5 | .411 | 0.918 | .09 | .46 | 3 | 1 |
| MT+ | 5 | .014* | 4.162 | .48 | 2.08 | 4 | 0 |
| combined | 5 | .151 | 1.773 | .07 | .89 | 3 | 0 |

**Table 13.** Statistics corresponding to Figure 3 - figure supplement 2, on pRF eccentricity versus x shift ratios. P-values result from t-tests over the 5 subject values. Single, double and triple asterisks indicate uncorrected p-value < .05, .01 and .001 respectively. The last two columns reflect in how many out of 5 subjects these differences were different from 0 with uncorrected bootstrapped p-values of <.05 over voxels.

| ROI | N | p | t | Δ ratio | Cohen's d | ecc>x | ecc<x |
|---|---|---|---|---|---|---|---|
| V1 | 5 | .049* | 2.797 | .04 | 1.40 | 3 | 0 |
| V2 | 5 | .350 | 1.057 | .03 | .53 | 2 | 1 |
| V3 | 5 | .036* | 3.094 | .06 | 1.55 | 3 | 0 |
| hV4 | 5 | .037* | 3.072 | .07 | 1.54 | 3 | 0 |
| VO | 5 | .013* | 4.240 | .08 | 2.12 | 5 | 0 |
| LO | 5 | .153 | 1.764 | .06 | .88 | 2 | 0 |
| V3AB | 5 | .037* | 3.084 | .14 | 1.54 | 4 | 0 |
| IPS0 | 5 | .047* | 2.845 | .15 | 1.42 | 4 | 0 |
| MT+ | 5 | .080 | 2.335 | .03 | 1.17 | 3 | 0 |
| combined | 5 | .003** | 6.707 | .05 | 3.35 | 5 | 0 |

**Table 14.** Statistics corresponding to Figure 3. Rayleigh test for non-uniformity for subjects 1-3. Single, double and triple asterisks indicate uncorrected p-value < .05, .01 and .001 respectively.

| s1 | s2 | s3 |
|---|---|---|

| ROI | N | z | p | N | z | p | N | z | p |
|---|---|---|---|---|---|---|---|---|---|
| V1 | 469 | 3.221 | .040* | 558 | 26.131 | <.001*** | 333 | 11.823 | <.001*** |
| V2 | 592 | 0.659 | .518 | 605 | 9.986 | <.001*** | 411 | 48.090 | <.001*** |
| V3 | 533 | 1.021 | .360 | 463 | 22.909 | <.001*** | 389 | 12.275 | <.001*** |
| hV4 | 335 | 18.664 | <.001*** | 183 | 0.626 | .535 | 228 | 32.801 | <.001*** |
| VO | 97 | 37.899 | <.001*** | 136 | 46.282 | <.001*** | 36 | 11.037 | <.001*** |
| LO | 271 | 2.255 | .105 | 367 | 25.638 | <.001*** | 268 | 37.663 | <.001*** |
| V3AB | 169 | 4.560 | .010* | 176 | 23.160 | <.001*** | 233 | 43.613 | <.001*** |
| IPS0 | 67 | 0.101 | .905 | 77 | 17.517 | <.001*** | 78 | 38.176 | <.001*** |
| MT+ | 43 | 14.066 | <.001*** | 71 | 17.095 | <.001*** | 43 | 27.463 | <.001*** |
| combined | 2576 | 12.585 | <.001*** | 2636 | 119.207 | <.001*** | 2019 | 208.441 | <.001*** |

**Table 15.** Statistics corresponding to Figure 3. Rayleigh test for non-uniformity for subjects 4-5. Single, double and triple asterisks indicate uncorrected p-value < .05, .01 and .001 respectively.

| | s4 | | | s5 | | |
|---|---|---|---|---|---|---|
| ROI | N | z | p | N | z | p |
| V1 | 369 | 3.891 | .020* | 447 | 40.575 | <.001*** |
| V2 | 563 | 4.195 | .015* | 583 | 20.792 | <.001*** |
| V3 | 504 | 22.465 | <.001*** | 433 | 27.032 | <.001*** |
| hV4 | 218 | 0.995 | .370 | 237 | 30.059 | <.001*** |
| VO | 146 | 24.882 | <.001*** | 167 | 28.314 | <.001*** |
| LO | 195 | 4.618 | .010* | 304 | 24.695 | <.001*** |
| V3AB | 176 | 3.471 | .031* | 129 | 18.098 | <.001*** |
| IPS0 | 49 | 12.339 | <.001*** | 45 | 11.335 | <.001*** |
| MT+ | 47 | 26.131 | <.001*** | 124 | 50.139 | <.001*** |
| combined | 2267 | 51.292 | <.001*** | 2469 | 163.225 | <.001*** |

**Table 16.** Statistics corresponding to Figure 4 - figure supplement 1, on pRF eccentricity changes in eccentricity bin 1. P-values result from t-tests against 0 over the 5 subject values. Single, double and triple asterisks indicate uncorrected significance of <.05, <.01 and <.001 respectively. Right most columns indicate in how many out of 5 subjects bootstrap tests over voxels were different from 0 with uncorrected p-value of < .05.

| ROI | N | p | t | Δ ecc | Cohen's d | pos in | neg in |
|---|---|---|---|---|---|---|---|
| V1 | 5 | .172 | -1.663 | -0.035 | -0.831 | 1 | 4 |
| V2 | 5 | .998 | 0.003 | 0.000 | 0.001 | 2 | 2 |
| V3 | 5 | .042* | 2.953 | 0.053 | 1.477 | 4 | 0 |
| hV4 | 5 | .052 | 2.729 | 0.124 | 1.365 | 5 | 0 |

| | | | | | | | |
|---|---|---|---|---|---|---|---|
| VO | 5 | .017* | 3.903 | 0.393 | 1.952 | 5 | 0 |
| LO | 5 | .030* | 3.309 | 0.125 | 1.655 | 5 | 0 |
| V3AB | 5 | .047* | 2.843 | 0.442 | 1.422 | 5 | 0 |
| IPS0 | 5 | .080 | 2.335 | 0.715 | 1.167 | 5 | 0 |
| MT+ | 5 | .089 | 2.237 | 0.858 | 1.119 | 5 | 0 |
| combined | 5 | .015* | 4.067 | 0.096 | 2.033 | 5 | 0 |

**Table 17.** Statistics corresponding to Figure 4 - figure supplement 1, on pRF eccentricity changes in eccentricity bin 2. P-values result from t-tests against 0 over the 5 subject values. Single, double and triple asterisks indicate uncorrected significance of <.05, <.01 and <.001 respectively. Right most columns indicate in how many out of 5 subjects bootstrap tests over voxels were different from 0 with uncorrected p-value of < .05.

| ROI | N | p | t | Δ ecc | Cohen’s d | pos in | neg in |
|---|---|---|---|---|---|---|---|
| V1 | 5 | .348 | -1.063 | -0.021 | -0.532 | 2 | 2 |
| V2 | 5 | .822 | 0.240 | 0.004 | 0.120 | 3 | 2 |
| V3 | 5 | .045* | 2.884 | 0.075 | 1.442 | 4 | 0 |
| hV4 | 5 | .049* | 2.805 | 0.216 | 1.402 | 5 | 0 |
| VO | 5 | .014* | 4.151 | 0.444 | 2.075 | 5 | 0 |
| LO | 5 | .087 | 2.252 | 0.491 | 1.126 | 5 | 0 |
| V3AB | 5 | .047* | 2.837 | 0.623 | 1.419 | 5 | 0 |
| IPS0 | 5 | .009** | 4.674 | 0.753 | 2.337 | 4 | 0 |
| MT+ | 5 | .075 | 2.391 | 1.463 | 1.195 | 5 | 0 |
| combined | 5 | .056 | 2.671 | 0.103 | 1.335 | 5 | 0 |

**Table 18.** Statistics corresponding to Figure 4 - figure supplement 1, on pRF eccentricity changes in eccentricity bin 3. P-values result from t-tests against 0 over the 5 subject values. Single, double and triple asterisks indicate uncorrected significance of <.05, <.01 and <.001 respectively. Right most columns indicate in how many out of 5 subjects bootstrap tests over voxels were different from 0 with uncorrected p-value of < .05.

| ROI | N | p | t | Δ ecc | Cohen’s d | pos in | neg in |
|---|---|---|---|---|---|---|---|
| V1 | 5 | .296 | -1.202 | -0.022 | -0.601 | 0 | 2 |
| V2 | 5 | .556 | -0.642 | -0.012 | -0.321 | 1 | 3 |
| V3 | 5 | .057 | 2.651 | 0.081 | 1.326 | 4 | 0 |
| hV4 | 5 | .001** | 8.314 | 0.319 | 4.157 | 5 | 0 |
| VO | 5 | .028* | 3.355 | 0.897 | 1.678 | 5 | 0 |
| LO | 5 | .120 | 1.969 | 0.444 | 0.985 | 4 | 0 |
| V3AB | 5 | .053 | 2.714 | 0.557 | 1.357 | 5 | 0 |
| IPS0 | 5 | .007** | 5.103 | 1.234 | 2.551 | 5 | 0 |

| | | | | | | | |
|---|---|---|---|---|---|---|---|
| MT+ | 4 | .016* | 4.896 | 1.378 | 2.827 | 4 | 0 |
| combined | 5 | .019* | 3.799 | 0.044 | 1.900 | 5 | 0 |

**Table 19.** Statistics corresponding to Figure 4 - figure supplement 1, on pRF eccentricity changes in eccentricity bin 4. P-values result from t-tests against 0 over the 5 subject values. Single, double and triple asterisks indicate uncorrected significance of <.05, <.01 and <.001 respectively. Right most columns indicate in how many out of 5 subjects bootstrap test over voxels were different from 0 with uncorrected p-value of < .05.

| ROI | N | p | t | Δ ecc | Cohen's d | pos in | neg in |
|---|---|---|---|---|---|---|---|
| V1 | 5 | .369 | -1.011 | -0.016 | -0.506 | 0 | 3 |
| V2 | 5 | .071 | -2.442 | -0.059 | -1.221 | 0 | 3 |
| V3 | 5 | .085 | -2.273 | -0.063 | -1.136 | 0 | 3 |
| hV4 | 5 | .082 | 2.308 | 0.202 | 1.154 | 1 | 0 |
| VO | 5 | .417 | 0.904 | 0.623 | 0.452 | 3 | 1 |
| LO | 5 | .285 | 1.234 | 0.782 | 0.617 | 2 | 1 |
| V3AB | 5 | .122 | 1.960 | 0.154 | 0.980 | 1 | 0 |
| IPS0 | 5 | .516 | 0.711 | 0.533 | 0.356 | 2 | 1 |
| MT+ | 4 | .187 | 1.590 | 1.662 | 0.795 | 2 | 1 |
| combined | 5 | .273 | -1.270 | -0.031 | -0.635 | 1 | 3 |

**Table 20.** Statistics corresponding to Figure 4 - figure supplement 1, on pRF size changes in eccentricity bin 1. P-values result from t-tests against 0 over the 5 subject values. Single, double and triple asterisks indicate uncorrected significance of <.05, <.01 and <.001 respectively. Right most columns indicate in how many out of 5 subjects bootstrap test over voxels were different from 0 with uncorrected p-value of < .05.

| ROI | N | p | t | Δ ecc | Cohen's d | pos in | neg in |
|---|---|---|---|---|---|---|---|
| V1 | 5 | .768 | 0.315 | 0.002 | 0.158 | 1 | 1 |
| V2 | 5 | .511 | -0.721 | -0.003 | -0.361 | 1 | 1 |
| V3 | 5 | .777 | 0.304 | 0.002 | 0.152 | 2 | 0 |
| hV4 | 5 | .934 | -0.088 | -0.002 | -0.044 | 1 | 2 |
| VO | 5 | .109 | 2.056 | 0.134 | 1.028 | 3 | 0 |
| LO | 5 | .671 | 0.457 | 0.016 | 0.228 | 2 | 2 |
| V3AB | 5 | .041* | 2.977 | 0.184 | 1.489 | 4 | 0 |
| IPS0 | 5 | .031* | 3.267 | 0.725 | 1.633 | 5 | 0 |
| MT+ | 5 | .124 | 1.943 | 0.603 | 0.971 | 4 | 0 |
| combined | 5 | .426 | 0.884 | 0.018 | 0.442 | 2 | 1 |

**Table 21.** Statistics corresponding to Figure 4 - figure supplement 1, on pRF size changes in eccentricity bin 2. P-values result from t-tests against 0 over the 5 subject values. Single, double and triple asterisks indicate uncorrected significance of <.05, <.01 and <.001 respectively. Right most columns indicate in how many out of 5 subjects bootstrap tests over voxels were different from 0 with uncorrected p-value of < .05.

| ROI | N | p | t | Δ ecc | Cohen's d | pos in | neg in |
|---|---|---|---|---|---|---|---|
| V1 | 5 | .572 | 0.614 | 0.003 | 0.307 | 2 | 1 |
| V2 | 5 | .697 | 0.418 | 0.002 | 0.209 | 2 | 1 |
| V3 | 5 | .937 | 0.085 | 0.000 | 0.042 | 2 | 1 |
| hV4 | 5 | .082 | 2.308 | 0.024 | 1.154 | 3 | 0 |
| VO | 5 | .037* | 3.089 | 0.078 | 1.544 | 3 | 0 |
| LO | 5 | .214 | 1.475 | 0.101 | 0.738 | 4 | 0 |
| V3AB | 5 | .033* | 3.211 | 0.175 | 1.605 | 5 | 0 |
| IPS0 | 5 | .027* | 3.419 | 0.367 | 1.709 | 4 | 0 |
| MT+ | 5 | .058 | 2.631 | 0.656 | 1.316 | 5 | 0 |
| combined | 5 | .088 | 2.250 | 0.015 | 1.125 | 4 | 1 |

**Table 22.** Statistics corresponding to Figure 4 - figure supplement 1, on pRF size changes in eccentricity bin 3. P-values result from t-tests against 0 over the 5 subject values. Single, double and triple asterisks indicate uncorrected significance of <.05, <.01 and <.001 respectively. Right most columns indicate in how many out of 5 subjects bootstrap tests over voxels were different from 0 with uncorrected p-value of < .05.

| ROI | N | p | t | Δ ecc | Cohen's d | pos in | neg in |
|---|---|---|---|---|---|---|---|
| V1 | 5 | .061 | 2.579 | 0.009 | 1.290 | 3 | 0 |
| V2 | 5 | .373 | -1.001 | -0.005 | -0.501 | 0 | 1 |
| V3 | 5 | .111 | -2.041 | -0.017 | -1.020 | 0 | 3 |
| hV4 | 5 | .376 | 0.995 | 0.022 | 0.498 | 1 | 0 |
| VO | 5 | .773 | 0.308 | 0.031 | 0.154 | 3 | 1 |
| LO | 5 | .800 | -0.271 | -0.007 | -0.135 | 1 | 0 |
| V3AB | 5 | .085 | 2.280 | 0.068 | 1.140 | 3 | 0 |
| IPS0 | 5 | .034* | 3.164 | 0.297 | 1.582 | 4 | 0 |
| MT+ | 4 | .076 | 2.662 | 0.285 | 1.537 | 2 | 0 |
| combined | 5 | .800 | -0.270 | -0.002 | -0.135 | 1 | 2 |

**Table 23.** Statistics corresponding to Figure 4 - figure supplement 1, on pRF size changes in eccentricity bin 4. P-values result from t-tests against 0 over the 5 subject values. Single, double and triple asterisks indicate uncorrected significance of <.05, <.01 and <.001 respectively. Right most columns indicate in

how many out of 5 subjects bootstrap tests over voxels were different from 0 with uncorrected p-value of < .05.

| ROI | N | p | t | Δ ecc | Cohen's d | pos in | neg in |
|---|---|---|---|---|---|---|---|
| V1 | 5 | .800 | 0.270 | 0.002 | 0.135 | 1 | 1 |
| V2 | 5 | .122 | -1.959 | -0.021 | -0.979 | 0 | 3 |
| V3 | 5 | .028* | -3.386 | -0.058 | -1.693 | 0 | 4 |
| hV4 | 5 | .188 | -1.587 | -0.141 | -0.793 | 0 | 3 |
| VO | 5 | .329 | -1.110 | -0.338 | -0.555 | 0 | 3 |
| LO | 5 | .997 | -0.004 | 0.000 | -0.002 | 0 | 1 |
| V3AB | 5 | .352 | -1.052 | -0.075 | -0.526 | 0 | 4 |
| IPS0 | 5 | .827 | -0.233 | -0.079 | -0.117 | 2 | 1 |
| MT+ | 5 | .257 | 1.322 | 0.349 | 0.661 | 2 | 0 |
| combined | 5 | .071 | -2.447 | -0.030 | -1.223 | 0 | 4 |

**Table 24.** Statistics corresponding to Figure 4 - figure supplement 2, on correlations between eccentricity and size changes. P-values result from t-tests against 0 over the 5 subject correlation values. Single and triple asterisks indicate uncorrected significance of <.01 and <.001 respectively. Right most columns indicate in how many out of 5 subjects bootstrap tests over voxels were different from 0 with uncorrected p-value of < .05.

| ROI | R | N | t | p | pos in | neg in |
|---|---|---|---|---|---|---|
| V1 | 0.461 | 5 | 2.888 | .045* | 2 | 0 |
| V2 | 0.704 | 5 | 6.411 | .003** | 4 | 0 |
| V3 | 0.704 | 5 | 7.219 | .002** | 5 | 0 |
| hV4 | 0.749 | 5 | 10.609 | <.001*** | 5 | 0 |
| VO | 0.886 | 5 | 12.673 | <.001*** | 5 | 0 |
| LO | 0.847 | 5 | 8.074 | .001** | 5 | 0 |
| V3AB | 0.861 | 5 | 6.954 | .002** | 5 | 0 |
| IPS0 | 0.500 | 5 | 2.450 | .070 | 3 | 0 |
| MT+ | 0.745 | 5 | 6.261 | .003** | 4 | 0 |
| combined | 0.799 | 5 | 5.431 | .006** | 4 | 0 |

**Table 25.** Statistics corresponding to Figure 6 - figure supplement 3. Asterisks indicate whether the average feature AMI over subjects was different from 0 with p < .05. Right most columns indicate in how many out of 5 subjects bootstrap tests over voxels were different from 0 with uncorrected p-value of < .05.

| ROI | N | mean diff | t | p | Cohen's | pos in | neg in |
|---|---|---|---|---|---|---|---|
| V1 | 5 | 0.062 | 3.111 | 0.036* | 1.555 | 4 | 0 |
| V2 | 5 | 0.046 | 3.315 | 0.030* | 1.657 | 4 | 0 |

| | | | | | | | |
|---|---|---|---|---|---|---|---|
| V3 | 5 | 0.025 | 1.981 | 0.119 | 0.991 | 3 | 0 |
| hV4 | 5 | 0.062 | 2.845 | 0.047* | 1.423 | 4 | 0 |
| VO | 5 | 0.080 | 1.443 | 0.222 | 0.722 | 2 | 0 |
| LO | 5 | 0.026 | 1.497 | 0.209 | 0.748 | 2 | 0 |
| V3AB | 5 | 0.090 | 3.208 | 0.033* | 1.604 | 4 | 0 |
| IPS0 | 5 | 0.092 | 1.584 | 0.188 | 0.792 | 2 | 0 |
| MT+ | 5 | 0.044 | 1.369 | 0.243 | 0.684 | 2 | 0 |
| comb | 5 | 0.047 | 4.316 | 0.012* | 2.158 | 4 | 0 |

**Table 26.** Statistics corresponding to Figure 7 - figure supplement 1. Asterisks indicate whether the spearman correlation between feature preference and eccentricity over subjects was different from 0 with $p < .05$. Right most columns indicate in how many out of 5 subjects bootstrap tests over voxels were different from 0 with uncorrected p-value of $< .05$.

| ROI | N | corr | p | pos in | neg in |
|---|---|---|---|---|---|
| V1 | 5 | -0.218 | .031* | 0 | 5 |
| V2 | 5 | -0.210 | .090 | 0 | 3 |
| V3 | 5 | -0.181 | .084 | 0 | 4 |
| hV4 | 5 | -0.222 | .095 | 0 | 3 |
| VO | 5 | -0.294 | .088 | 0 | 3 |
| LO | 5 | -0.133 | .138 | 0 | 4 |
| V3AB | 5 | -0.250 | .105 | 1 | 3 |
| IPS0 | 5 | -0.183 | .076 | 0 | 2 |
| MT+ | 5 | -0.273 | .085 | 0 | 2 |
