# Supplementary material for: Spatial sampling in human visual cortex is modulated by both spatial and feature-based attention
Source: eLife. 2018 Dec 7;7:e36928. doi: 10.7554/eLife.36928 (PMC6286128; doi:10.7554/eLife.36928)
Supplement: Transparent reporting form [file elife-36928-transrepform.docx]

***eLife’s* transparent reporting form**

We encourage authors to provide detailed information *within their submission* to facilitate the interpretation and replication of experiments. Authors can upload supporting documentation to indicate the use of appropriate reporting guidelines for health-related research (see [EQUATOR Network](http://www.equator-network.org/%20)), life science research (see the [BioSharing Information Resource](https://biosharing.org/)), or the [ARRIVE guidelines](http://www.plosbiology.org/article/info:doi/10.1371/journal.pbio.1000412) for reporting work involving animal research. Where applicable, authors should refer to any relevant reporting standards documents in this form.

If you have any questions, please consult our Journal Policies and/or contact us: [editorial@elifesciences.org](mailto:editorial@elifesciences.org).

**Sample-size estimation**

- You should state whether an appropriate sample size was computed when the study was being designed
- You should state the statistical method of sample size computation and any required assumptions
- If no explicit power analysis was used, you should describe how you decided what sample (replicate) size (number) to use

Please outline where this information can be found within the submission (e.g., sections or figure legends), or explain why this information doesn’t apply to your submission:

This information can be found in the Methods section called ‘pRF parameter analyses’

**Replicates**

- You should report how often each experiment was performed
- You should include a definition of biological versus technical replication
- The data obtained should be provided and sufficient information should be provided to indicate the number of independent biological and/or technical replicates
- If you encountered any outliers, you should describe how these were handled
- Criteria for exclusion/inclusion of data should be clearly stated
- High-throughput sequence data should be uploaded before submission, with a private link for reviewers provided (these are available from both GEO and ArrayExpress)

Please outline where this information can be found within the submission (e.g., sections or figure legends), or explain why this information doesn’t apply to your submission:

The number of experimental runs for each experiment and participant is stated under the Methods section called ‘MRI acquisition’. These multiple recordings are meant to reduce both technical and biological sources of noise without explicitly separating them. We therefore believe that explicit definitions of biological and technical replications do not apply to our submission.

The description of outlier handling can be found in the Methods section called ‘pRF parameter analyses’.

**Statistical reporting**

- Statistical analysis methods should be described and justified
- Raw data should be presented in figures whenever informative to do so (typically when N per group is less than 10)
- For each experiment, you should identify the statistical tests used, exact values of N, definitions of center, methods of multiple test correction, and dispersion and precision measures (e.g., mean, median, SD, SEM, confidence intervals; and, for the major substantive results, a measure of effect size (e.g., Pearson's r, Cohen's d)
- Report exact p-values wherever possible alongside the summary statistics and 95% confidence intervals. These should be reported for all key questions and not only when the p-value is less than 0.05.

Please outline where this information can be found within the submission (e.g., sections or figure legends), or explain why this information doesn’t apply to your submission:

The description and justification of statistical tests used for each analysis can be found under the section ‘pRF parameter analyses’.

Source data is available for download (for links see box under **Additional data files(“source data”)**).

All descriptive and inferential statistics can be found in the statistical tables provided as a separate appendix at the end of our manuscript. This includes the method of multiple test correction, N, absolute and statistical effect sizes and exact p-values.

95% confidence intervals are reported within the figures as error bars.

(For large datasets, or papers with a very large number of statistical tests, you may upload a single table file with tests, Ns, etc., with reference to sections in the manuscript.)

**Group allocation**

- Indicate how samples were allocated into experimental groups (in the case of clinical studies, please specify allocation to treatment method); if randomization was used, please also state if restricted randomization was applied
- Indicate if masking was used during group allocation, data collection and/or data analysis

Please outline where this information can be found within the submission (e.g., sections or figure legends), or explain why this information doesn’t apply to your submission:

Our experiments comprise within-subject designs. This means that all subjects performed all conditions and thus no allocation into experimental groups was required.

**Additional data files (“source data”)**

- We encourage you to upload relevant additional data files, such as numerical data that are represented as a graph in a figure, or as a summary table
- Where provided, these should be in the most useful format, and they can be uploaded as “Source data” files linked to a main figure or table
- Include model definition files including the full list of parameters used
- Include code used for data analysis (e.g., R, MatLab)
- Avoid stating that data files are “available upon request”

Please indicate the figures or tables for which source data files have been provided:

The source data for all figures, tables and analyses presented within the text can be found at <https://doi.org/10.6084/m9.figshare.c.4012717.v1>. The analyses code can be found under: <http://www.github.com/daanvanes/PRF_attention_analysis>

The data file includes all model parameters pertaining to the pRF and feature GLM fits.

We’ve included a link to the analysis code in the Methods section. The README in this analysis repository links to specific files within the Figshare repository.

**References**

Dumoulin, S.O. & Wandell, B.A., 2008. Population receptive field estimates in human visual cortex. *NeuroImage*, 39(2), pp.647–660.

Kay, K.N., Weiner, K.S. & Grill-Spector, K., 2015. Attention reduces spatial uncertainty in human ventral temporal cortex. *Current biology : CB*, 25(5), pp.595–600.

Klein, B.P., Harvey, B.M. & Dumoulin, S.O., 2014. Attraction of position preference by spatial attention throughout human visual cortex. *Neuron*, 84(1), pp.227–237.

Poldrack, R.A., 2017. Precision neuroscience: Dense sampling of individual brains. *Neuron*, 95(4), pp.727–729.
